# Endobronchial Carcinoid Tumour with Extensive Ossification: An Unusual Case Presentation

**DOI:** 10.1155/2016/5984671

**Published:** 2016-08-16

**Authors:** Allison Osmond, Emily Filter, Mariamma Joseph, Richard Inculet, Keith Kwan, David McCormack

**Affiliations:** ^1^Department of Anatomic Pathology, Western University, London, ON, Canada N6A 5C1; ^2^Department of Anatomic Pathology, Dalhousie University, Halifax, NS, Canada B3H 4R2; ^3^Department of Thoracic Surgery, Western University, London, ON, Canada N6A 5C1; ^4^Department of Medicine, Western University, London, ON, Canada N6A 5C1

## Abstract

Carcinoid tumour is a well-known primary endobronchial lung neoplasm. Although calcifications may be seen in up to 30% of pulmonary carcinoid tumours, near complete ossification of these tumours is an unusual finding. Such lesions can prove diagnostically challenging at the time of intraoperative frozen section as the latter technique requires thin sectioning of the lesion for microscopic assessment. We present an unusual case of endobronchial carcinoid tumour with extensive ossification in a 45-year-old male. Preliminary intraoperative diagnosis was achieved through the alternative use of cytology scrape smears. The final diagnosis was confirmed after decalcification of the tumour. The prognostic implications of heavily ossified carcinoid tumours remain elusive. Long-term clinical follow-up of these patients is recommended.

## 1. Case Presentation

A 45-year-old male presented to the Emergency Department with two episodes of hemoptysis and progressive exertional wheezing over a 9-month period. The patient's past medical history was unremarkable apart from a previous 20 pack-year smoking history. He reported a single episode of hemoptysis 8 years priorly and was lost to follow-up after initial radiologic investigations identified a 3.1 cm mass. He denied any interim respiratory symptoms.

**Figure 1:**
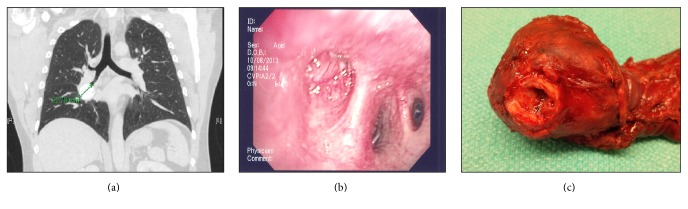
(a) CT thorax, coronal slice demonstrating a radiopaque hilar mass; (b) the hypervascular mass, as seen under bronchoscopic exam, occluding the bronchus; (c) the right middle lobe resection specimen. The mass encases the bronchial resection margin. The fleshy, endobronchial portion (*∗*) was sampled at the time of frozen section.

On physical exam, the patient had normal vital signs and was in no apparent distress. His chest exam was normal and there were no stigmata of chronic lung disease. There was no palpable lymphadenopathy. Chest CT imaging revealed a calcified 4.2 cm hilar mass with associated right middle lobe collapse (Figure 1(a)). The radiologic differential diagnosis included granuloma, carcinoid tumour, or benign nodule. Bronchoscopy confirmed the presence of a highly vascular and obstructive endobronchial mass (Figure 1(b)). Fine needle aspiration (FNA) of the mass was indeterminate due to low cellularity, despite multiple aspiration attempts. Due to the bronchoscopic appearance, the tumor was presumed to be a carcinoid tumor and so a thoracic surgical opinion was sought for consideration of resection. A right bilobectomy was then undertaken with an intraoperative consultation for preliminary diagnosis and to determine the bronchial resection margin status.

**Figure 2:**
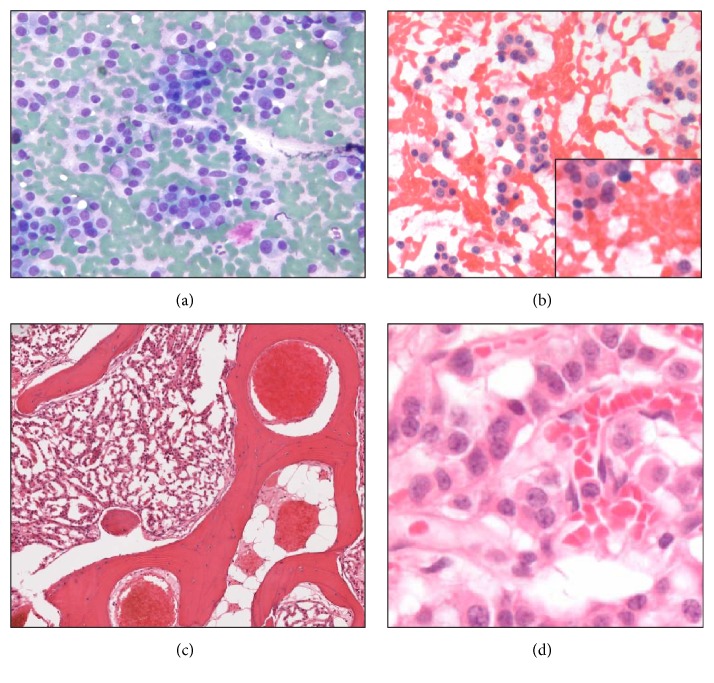
((a) and (b)) Cytology scrape smears including Diff Quik, 20x (c) and H&E, 20x (inset 40x); (c) H&E section of calcified mass demonstrating mature lamellar bone and nests of tumor cells; (d) trabecular structures and vague rosettes were noted (40x); the tumor cells were diffusely positive for chromogranin positivity, synaptophysin, and cytokeratin AE1/3.

Intraoperative gross specimen assessment revealed a hard, well-circumscribed, ossified 5.0 cm mass (Figure 1(c)) encasing the bronchial resection margin. The firm consistency of the mass precluded conventional tissue sectioning for frozen section evaluation. Cytology scrape smears (Diff Quik, H&E stains) were then prepared from a fleshy endobronchial portion of the mass. The smears contained a uniform population of tumour cells disposed singly and in loosely cohesive groups and rosettes (Figure 2(a)). The H&E scrape smear preparation confirmed the presence of a stippled “salt and pepper” chromatin pattern, characteristic of low grade neuroendocrine tumours (Figure 2(b)). A shave section of the bronchial resection margin was negative for malignancy. The preliminary diagnosis at intraoperative consultation was favoured to be carcinoid tumour with the final diagnosis deferred to permanent sections.

Microscopic examination of the formalin-fixed, decalcified mass revealed mature, anastomosing trabecular bone, with intervening nests of uniform tumour cells. The cells were occasionally arranged in trabecular structures and vague rosettes (Figures 2(c) and 2(d)). Despite a prolonged decalcification process, the tumour cells were strongly positive for chromogranin, synaptophysin, and cytokeratin AE1/AE3 immunohistochemistry markers. Necrosis and proliferative activity were absent (Ki-67 index < 1%). Two associated hilar lymph nodes were negative for metastasis. The final pathologic stage was pT2aN0MX.

## 2. Discussion

The WHO classification recognizes 4 histologic types of pulmonary neuroendocrine tumours: typical carcinoids, atypical carcinoids, small cell carcinomas, and large cell neuroendocrine carcinomas [1]. The typical carcinoid is a well-known primary endobronchial lung neoplasm accounting for 80–90% of pulmonary carcinoid tumours [1]. Most pulmonary carcinoids are identified incidentally as centrally located, well-circumscribed masses with routine imaging modalities.

Clinically, pulmonary carcinoids occur more frequently in males and typically manifest in the 5th decade of life [2]. In symptomatic patients, the most common symptoms include cough, hemoptysis, and a new-onset inspiratory wheeze [2]. Development of Cushing's syndrome secondary to ectopic ACTH production is uncommon [1]. At the time of bronchoscopy, pulmonary carcinoids can be accurately identified given their characteristic location and highly vascular appearance [2]. Nevertheless, cytologic and tissue diagnosis can be achieved by taking samples under bronchoscopic visualization with appropriate hemostatic control. If an intraoperative consultation is requested for preliminary tissue diagnosis, pulmonary carcinoid tumours can be suspected based on their characteristic solid and yellow gross appearance [1]. Frozen section examination allows an accurate diagnosis of low grade neuroendocrine neoplasms. However, in our case, extensive calcification precluded routine frozen section examination and cytology scrape smears greatly assisted in determining both the preliminary diagnosis and patient treatment plan.

As many as 30% of typical carcinoids may be accompanied by intralesional dystrophic calcifications [3] while complete ossification of these tumours is an unusual finding scarcely reported in the literature [3–6]. Ossification is more commonly associated with tumours of long duration [7], congruent with our case presentation. “Osteomimicry,” or the ability of tumour cells to upregulate osteogenic and osteoblastic gene expression (e.g., bone morphogenetic protein, osteocalcin) and thus acquire an osseous phenotype, is believed to play a role in ossification of pulmonary carcinoids [2]. Osteomimicry is well characterized in “bone prone” epithelial tumours (e.g., breast and prostate carcinomas) [8, 9]. This ability is believed to be advantageous as it augments epithelial-mesenchymal transition, permitting tumour cells to seed and survive in bony environments, similar to advanced breast and prostate carcinomas [9]. Moreover, a subset of patients with ossified pulmonary carcinoid tumours have presented with concomitant lymph node metastasis [3]. Whether or not osteomimicry in carcinoid tumors portends a more robust metastatic potential is unknown given the scarce number of cases reported in the literature. In our case, lymph node disease was absent at presentation and our patient remains free of metastatic disease 8 months postoperatively.

In summary, although extensive ossification is an unusual presentation for pulmonary carcinoid tumours, the implication of this phenotype remains elusive. Long-term follow-up of these patients is required to better characterize this phenomenon in disease progression. Finally, this case highlights the utility of cytology scrape preparations in making a diagnosis at the time of intraoperative consultation when specimens may not be suitable for usual frozen section technique.

## Learning Objectives

 Recognize the rare presentation of ossification in pulmonary carcinoids.
 Realize the limits of intraoperative consultation in extensively ossified lung tumours.

## CanMEDS Competency

Medical Expert is considered.
